# Anatomical and mechanical properties of swine midpalatal suture in the premaxillary, maxillary, and palatine region

**DOI:** 10.1038/s41598-018-25402-y

**Published:** 2018-05-04

**Authors:** Fabio Savoldi, Bing Xu, James K. H. Tsoi, Corrado Paganelli, Jukka P. Matinlinna

**Affiliations:** 1 Dental Materials Science, Discipline of Applied Oral Sciences, Faculty of Dentistry, The University of Hong Kong, Pok Fu Lam, Hong Kong; 2 Department of Orthodontics, Dental School, University of Brescia, Brescia, Italy; 3 Orthodontics, Faculty of Dentistry, The University of Hong Kong, Pok Fu Lam, Hong Kong; 4 Dental Department, The University of Hong Kong-Shenzhen Hospital, Shenzhen, P.R. China

## Abstract

The mechanical properties of the midpalatal suture and their relationship with anatomical parameters are relevant for both tissue engineering and clinical treatments, such as in sutural distraction osteogenesis. Soft tissues were dissected from ten swine heads and the hard palate was sliced perpendicularly to the midpalatal suture. Thirteen specimens were collected from each animal and analysed with micro-computed tomography and 4-point-bending for sutural width (*Sw*), interdigitation (*LII*), obliteration (*LOI*), failure stress (*σ*_*f*_), elastic modulus (*E*), and bone mineral density (*BMD*). Values of the premaxillary, maxillary, and palatine region were compared with Kruskal-Wallis one-way ANOVA and Spearman’s rank coefficient was used to analyse the correlation between parameters and their position along the suture (α = 0.05). *LII* had values of 1.0, 2.9, and 4.3, *LOI* had values of 0.0%, 2.5%, and 4.5%, and *E* had values of 12.5 MPa, 31.3 MPa, and 98.5 MPa, in the premaxillary, maxillary, and palatine region, respectively (p < 0.05). Failure stress and rigidity of the midpalatal suture increased from rostral to caudal, due to greater interdigitation and obliteration. These anatomical and mechanical findings contribute to characterise maxillary growth, and may help to understand its mechanical reaction during loading, and in virtual simulations.

## Introduction

During growth, a system of joints develops at the interface of the adjacent bones of the skull, most of which are a type of syndesmosis described as “*suture*”. Sutures allow stress distribution and bone remodelling during growth and through sutural distraction osteogenesis, as documented in human^1,2^ and swine^3^. Histologically, human sutures are characterised by a fibrous connective tissue at the interface between the bony fronts *i.e*., the sutural ligament, composed of several cellular layers and fibres^4^, whose general structure is similar between man and species such as rabbit, sheep, and swine^4,5^.

In humans, the hard palate originates during the intramembranous development of the nasomaxillary complex, and is formed by two lateral processes that grow toward the median line, and one anterior part known as primary palate^6^. Eventually, the hard palate consists of three pairs of bones connected along the midline by the midpalatal suture. This is divided into a premaxillary, a maxillary, and a palatine segment. Subsequently, the midpalatal suture further undergoes morphological changes progressively until adulthood^7^.

Although animal studies have reported that the midpalatal suture is a type of viscoelastic material^8,9^, quantitative information is very sparse and research studies had to assume the suture to behave as an empty space^10^, or to attribute to it the characteristics of other tissues^11^. Furthermore, studies on humans suggested that mechanical properties may be affected by its rostro-caudal gradient of ossification^12,13^, that is a relationship apparently still undocumented. Its reaction to loading is also relevant for regenerative medicine since sutural distraction is capable in inducing osteogenesis^14^, whose potential has found application in clinical treatments such as the maxillary expansion^15,16^ *i.e*., the widening of the upper jaw along the midpalatal suture.

The objectives of this study were to assess the change in the morphology and mechanical properties along the midpalatal suture of the swine, testing if any difference was present among the three analysed regions *i.e*., the premaxillary, the maxillary, and the palatine, and if any correlation existed with the position along the rostro-caudal direction.

## Results

### Histology and scanning electron microscopy

**Figure 1:**
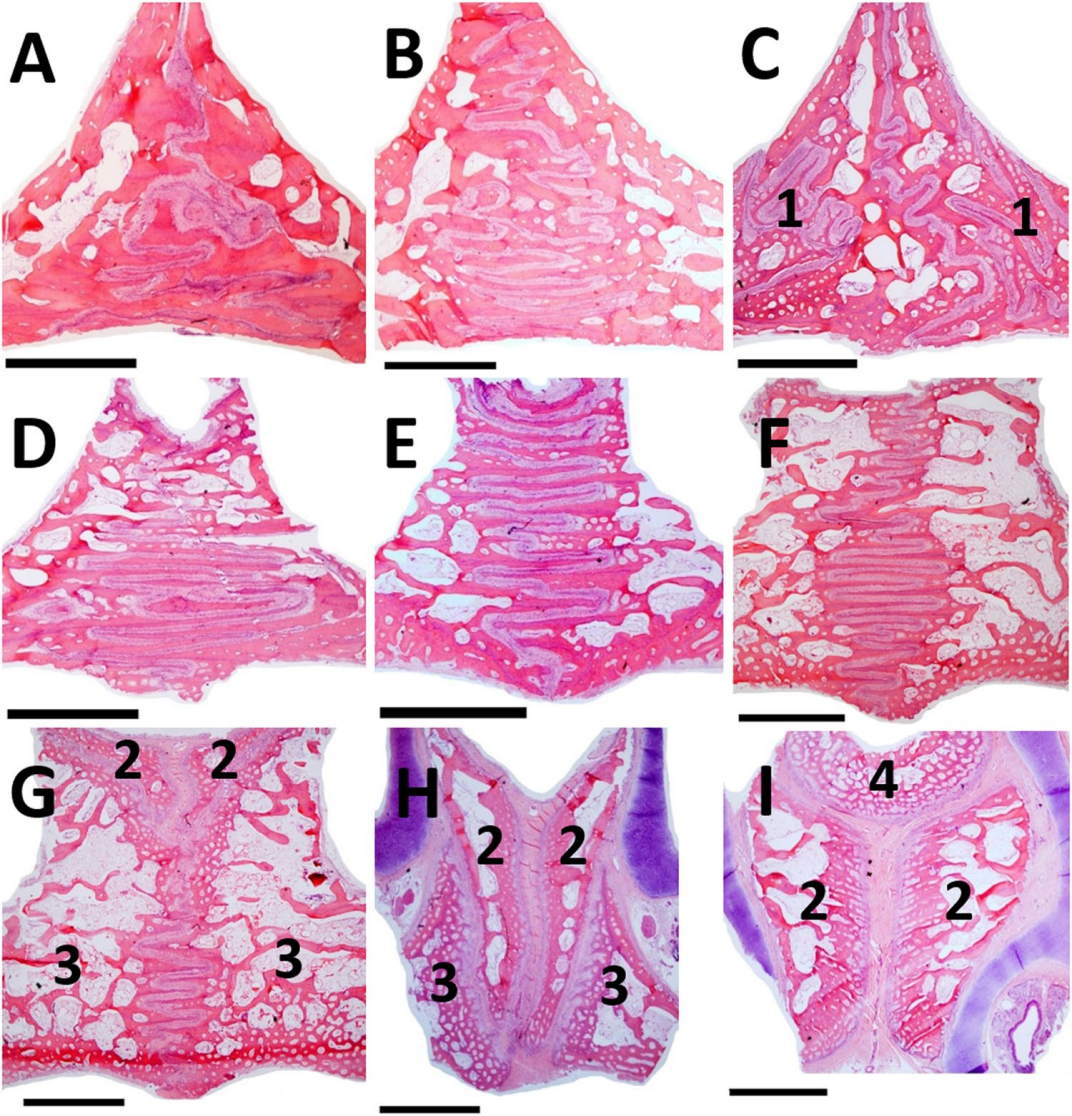
Histological images of the midpalatal suture (haematoxylin-eosin, 1×, bar = 2000 μm) on the coronal plane (rostro-caudal view). Irregular shape in the palato-palatine suture (**A**–**C**) and maxillo-palatal sutures on the sides (1); serrated pattern of the maxillo-maxillary suture (**D–F**); overlapping of different structures in the premaxillary region (**G**–**I**), including the premaxillo-premaxillary suture (2), the maxillo-maxillary suture (3), and the premaxillo-vomeral suture (4).

Proceeding from rostral to caudal, the general anatomy of the suture in the premaxillary region was a simple linear connection between the bony borders. At the most rostral part, a Y-shaped structure was visible, resulting from the overlapping of different structures. This included the straight connection of the premaxillo-premaxillary suture, the zig-zag pattern of the maxillo-maxillary suture, and the concave profile of the vomer inserting on the premaxilla (Fig. 1G–I). In the maxillary region the suture was progressively serrated, changing to an extended and parallel sinusoidal arrangement (Fig. 1D–F). Further, in the palatine region the sutural complexity was greater, with evident physical interlocking. The two maxillo-palatal sutures were also visible on its left and right side (Fig. 1A–C).

**Figure 2:**
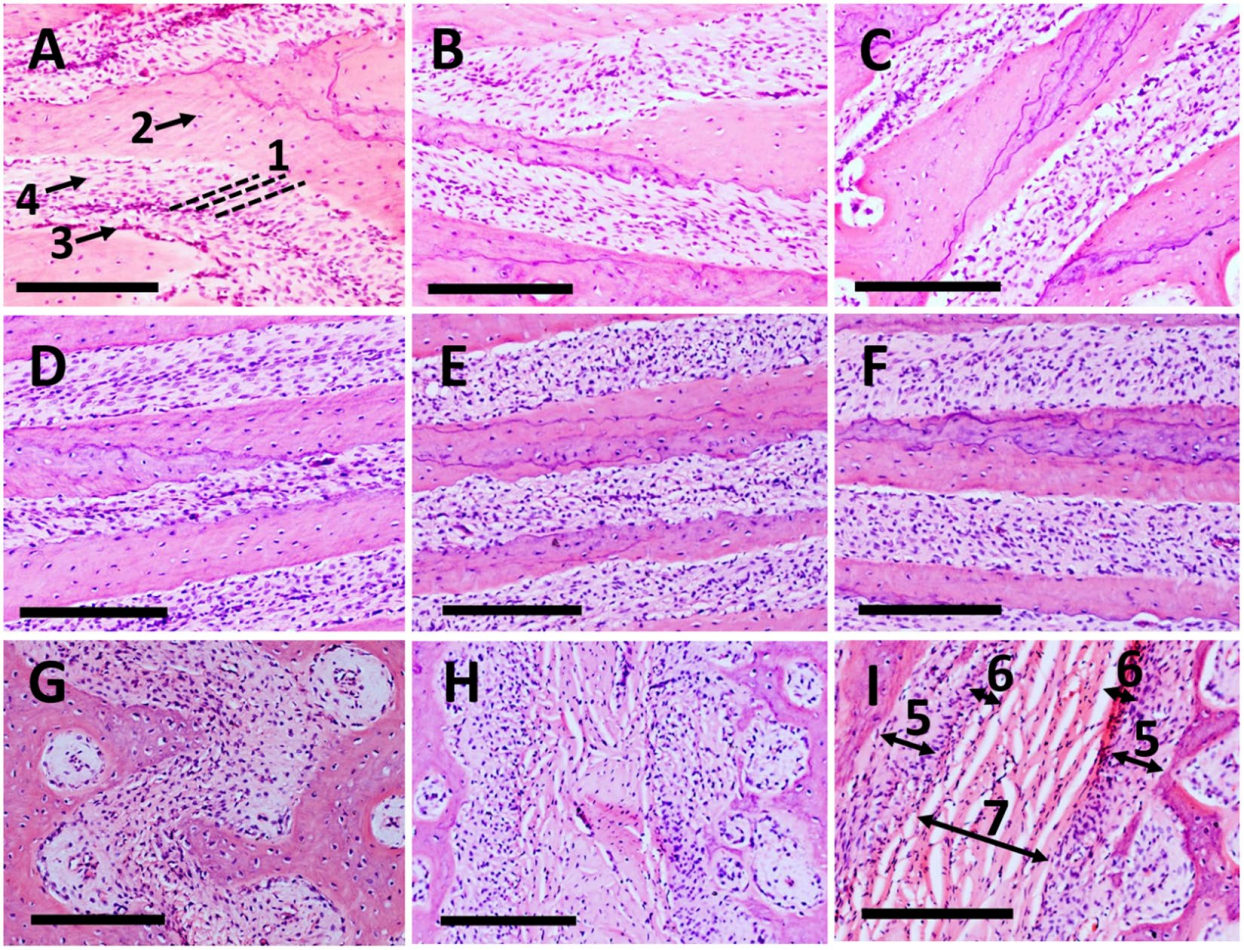
Histological images of the midpalatal suture (haematoxylin-eosin, 10×, bar = 200 μm) in the coronal plane (rostro-caudal view) from caudal (**A**) to rostral (**I**), following alphabetical order. In the most posterior region (**A**), the image shows an oblique arrangement of many ligament fibres connecting the opposing bony fronts (1); osteocytes-like cells in the inner part of the cortical bone (2); osteoblasts-like cells on the surface facing the sutural interface (3); and fibroblasts-like cells in the ligament (4). The rostral region presents two active cambial layers (5) and capsular layers (6), with a loose layer in the middle (7). Two distinct patterns of fibre orientation are visible: a parallel disposition in the most medial part of the suture, and a more radial orientation in contact with the bone (**I**).

The fibrous ligament of the suture presented five well-identifiable layers in the premaxillary region. In the most rostral part, cambial layers were thick, exhibiting several strata of proliferating osteogenic-like cells and appearing as growth sites. The capsular layers and the loose middle layer comprised of less cells and more fibres connecting the two sides. In this same region, two distinct patterns of fibre orientation were distinguishable: a parallel disposition in the most medial part, and a more radial orientation that was in contact with the bone (Fig. 2G–I). Compared to the premaxillary region, the ligament of the maxillo-maxillary suture was relatively dense with more cells, and the two distinct orientations of the fibres were not identifiable (Fig. 2D–F). The palatal region showed very thin cambial layers with a single layer of cells flattened against the bony surface. The capsular layers and the middle layer were not easily recognisable, forming the main bulk of the suture. The region showed more robust trabeculae and mature bone as indicated by the occasional presence of osteons-like cells (Fig. 2A–C). As a whole, the anatomy and the organisation of the sutural ligament of the premaxillary region were markedly different from the maxillary and the palatine.

**Figure 3:**
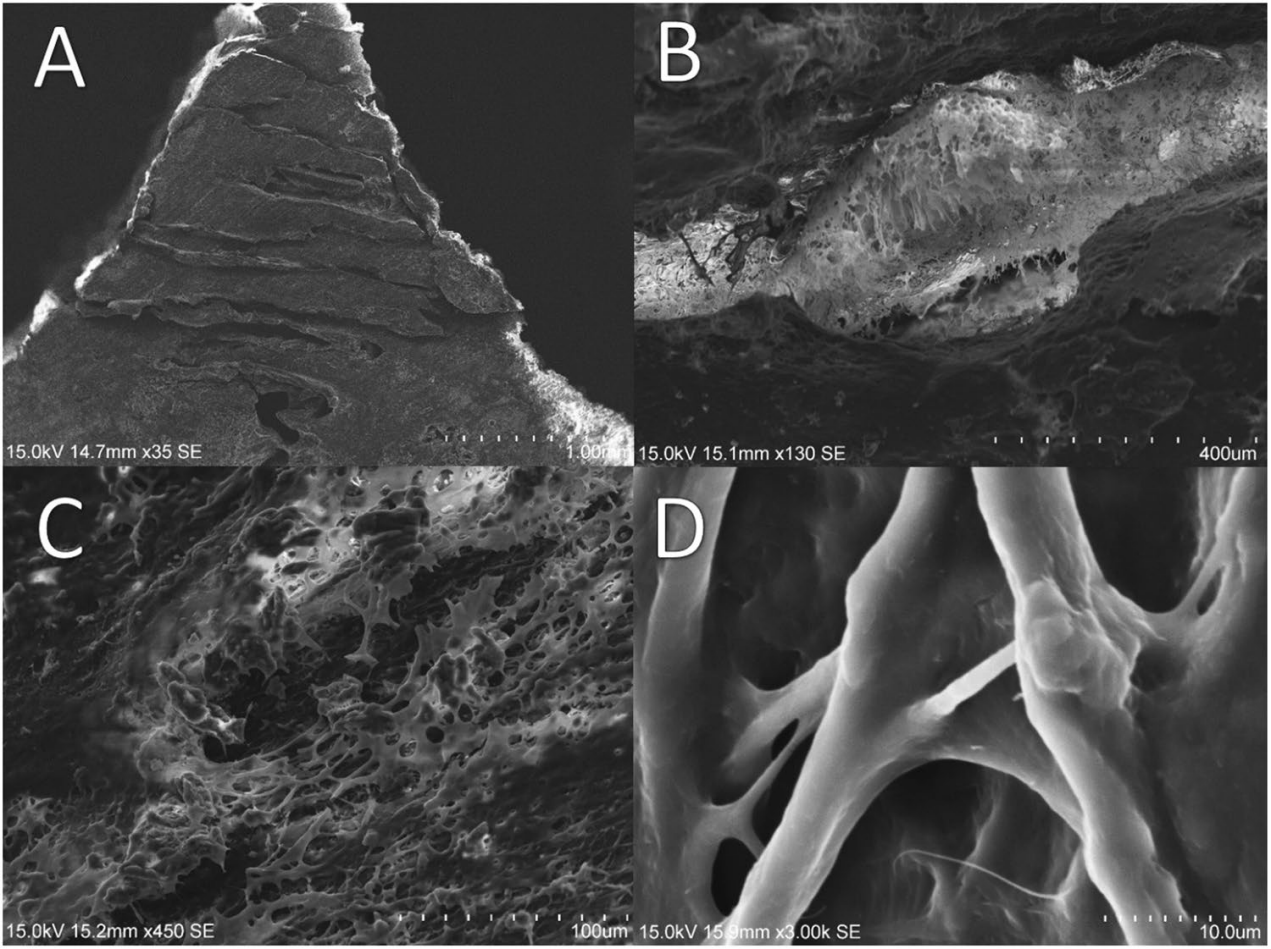
SEM images showing the coronal plane (rostro-caudal view) of a specimen from the maxillary region. The general view (35×) illustrates the highly interdigitated suture along the triangular-shaped bone with the base facing the oral cavity, and the apex oriented towards the nasal cavity for connection with the vomer (**A**); bone-ligament-bone interface (130×), (**B**); sutural ligament (450×) (**C**); detail of sutural ligament fibres (1000×) (**D**).

Scanning-electron microscopy (SEM) pictures corroborated the histological findings showing a complex sinusoidal interface composed of two opposing and relatively flat fronts facing each other at a distance in the order of hundreds of microns. An intricate matrix of tubular-like fibrillar strings of 1 to 10 µm of diameter emerged converging towards the medial region of the interface following different orientations. In addition, a smooth and relatively uniform layer covered the surface of the fibres, which were not distinguishable as single structures and appeared as a net-like pattern (Fig. 3).

### Anatomy

The data were not normally distributed (p < 0.05) and thus non-parametric tests were applied.

**Figure 4:**
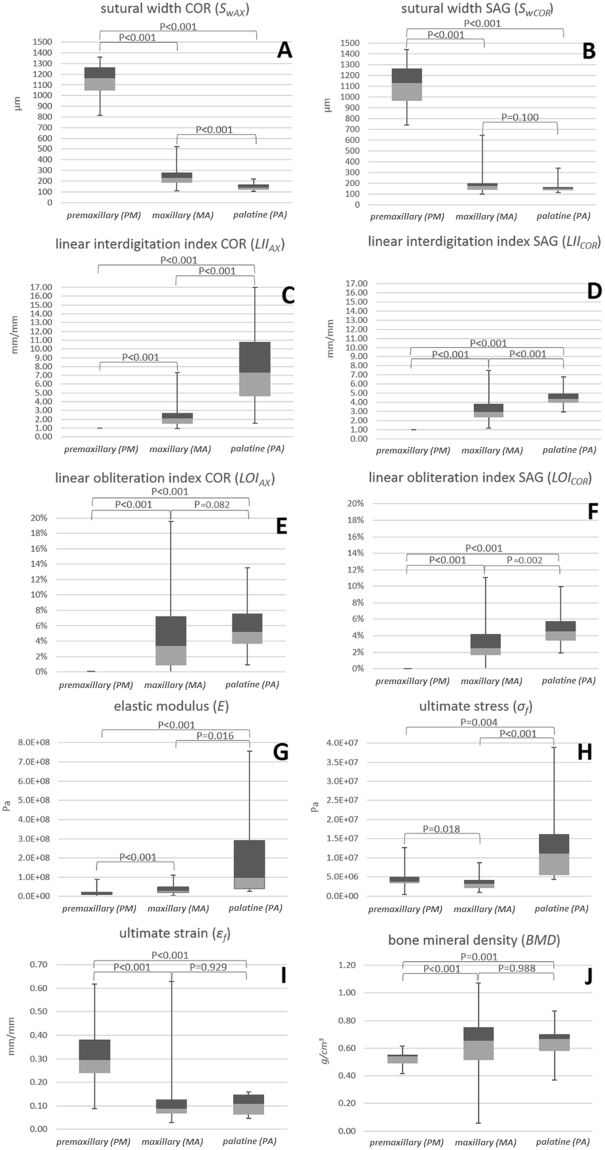
Sutural width (*Sw*) (**A** and **B**), linear interdigitation index (*LII*) (**C** and **D**), and linear obliteration index (*LOI*) (**E** and **F**) values in the axial (AX) and coronal (COR) plane. Elastic modulus (*E*) (**G**), failure stress (*σ*_*f*_) (**H**), failure strain (*ε*_*f*_) (**I**), bone mineral density (*BMD*) (**J**) values in the three analysed regions. Boxplot parameters: the top line of the upper box (dark grey) represents the 75^th^ percentile (3^rd^ quartile); the line between the upper box and the lower box represents the 50^th^ percentile (2^nd^ quartile); the bottom line of the lower box (light grey) represents the 25^th^ percentile (1^st^ quartile); the top of the upper whisker represents the maximum and the bottom of the lower whisker represents the minimum.

*Sw* had median values of 1162 µm (IQR = 218 µm) and 1132 µm (IQR = 300 µm) in the premaxillary region, 229 µm (IQR = 98 µm) and 172 µm (IQR = 59 µm) in the maxillary region, and 137 µm (IQR = 50 µm) and 148 µm (IQR = 28 µm) in the palatine, in the axial (*Sw*_*AX*_) and coronal (*Sw*_*COR*_) plane, respectively (Table 1, Fig. 4A,B). *Sw* showed a negative correlation with *Pos*_*CON*_ of −0.754 (p < 0.001) and −0.443 (p < 0.001), regarding the axial and coronal plane, respectively.

**Table 1:** Values of the anatomical and mechanical parameters.

			Premaxillary region (PM)	Maxillary region (MA)	Palatine region (PA)
*Sw*_*AX*_	µm	median	1162	229	137
		IQR	218	98	50
*Sw*_*COR*_	µm	median	1132	172	148
		IQR	300	59	28
*LII*_*AX*_	mm/mm	median	1.0	2.1	7.3
		IQR	0.0	1.2	6.2
*LII*_*COR*_	mm/mm	median	1.0	2.9	4.3
		IQR	0.0	1.5	1.0
*LOI*_*AX*_	%	median	0.0	3.4	5.2
		IQR	0.0	6.4	3.9
*LOI*_*COR*_	%	median	0.0	2.5	4.5
		IQR	0.0	2.6	2.3
*BMD*	g/cm^3^	median	0.54	0.65	0.67
		IQR	0.07	0.24	0.12
*E*	MPa	median	12.5	31.3	98.5
		IQR	11.2	29.6	250.3
*σ*_*f*_	MPa	median	3.8	3.2	11.1
		IQR	1.7	2.2	10.7
*ε*_*f*_	%	median	29.5	8.8	10.8
		IQR	14.3	6.0	8.5

IQR = interquartile range.

*LII* had median values of 1.0 (IQR = 0.0) and 1.0 (IQR = 0.0) in the premaxillary region, 2.1 (IQR = 1.2) and 2.9 (IQR = 1.5) in the maxillary region, and 7.3 (IQR = 6.2) and 4.3 (IQR = 1.0) in the palatine, in the axial (*LII*_*AX*_) and coronal (*LII*_*COR*_) plane, respectively (Table 1, Fig. 4C,D). *LII* showed a positive correlation with *Pos*_*CON*_ of +0.718 (p < 0.001) and +0.473 (p < 0.001), regarding the axial and coronal plane, respectively.

*LOI* had median values of 0.0% (IQR = 0.0%) and 0.0% (IQR = 0.0%) in the premaxillary region, 3.4% (IQR = 6.4%) and 2.5% (IQR = 2.6%) in the maxillary region, and 5.2% (IQR = 3.9%) and 4.5% (IQR = 2.3%) in the palatine, in the axial (*LOI*_*AX*_) and coronal (*LOI*_*COR*_) plane, respectively (Table 1, Fig. 4E,F). *LOI* showed a positive correlation with *Pos*_*CON*_ of +0.513 (p < 0.001) and +0.725 (p < 0.001), regarding the axial and coronal plane, respectively.

*BMD* had median values of 0.54 g/cm^3^ (IQR = 0.07 g/cm^3^) in the premaxillary region, 0.65 g/cm^3^ (IQR = 0.24 g/cm^3^) in the maxillary region, and 0.67 g/cm^3^ (IQR = 0.12 g/cm^3^) in the palatine (Fig. 4J). *BMD* showed a positive correlation with *Pos*_*CON*_ of +0.532 (p < 0.001).

In general, besides each inter-group comparison, the anatomy of the premaxillary region resulted to be remarkably different from the others.

### Mechanical properties

*E* had median values of 12.5 MPa (IQR = 11.2 MPa) in the premaxillary region, 31.3 MPa (IQR = 29.6 MPa) in the maxillary region, and 98.5 MPa (IQR = 250.3 MPa) in the palatine (Table 1 and Fig. 4G). *E* showed a positive correlation with *Pos*_*CON*_ of +0.494 (p < 0.001).

*σ*_*f*_ had median values of 3.8 MPa (IQR = 1.7 MPa) in the premaxillary region, 3.2 MPa (IQR = 2.2 MPa) in the maxillary region, and 11.1 MPa (IQR = 10.7 MPa) in the palatine (Table 1 and Fig. 4H). Although *σ*_*f*_ did not show a significant correlation with *Pos*_*CON*_ (+0.625, p = 0.144), it had significantly higher values in the palatine region.

*ε*_*f*_ had median values of 29.5% (IQR = 14.3%) in the premaxillary region, 8.8% (IQR = 6.0%) in the maxillary region, and 10.8% (IQR = 8.5%) in the palatine (Table 1 and Fig. 4I). *ε*_*f*_ showed a negative correlation with *Pos*_*CON*_ of −0.691 (p < 0.001).

## Discussion

### Histology and scanning electron microscopy

Of the Y-shaped articulation found in the premaxilla (Fig. 1G–I), only the lower and vertical part of the “Y” strictly belonged to the midpalatal suture, and the similar structure also described in humans^7,17^ should be considered as composed by multiple sutures. In fact, the two upper and oblique lines represented the premaxillo-vomeral suture (Fig. 1I) and, more posteriorly, they resulted from the overlapping of the caudal protrusion of the premaxilla on the maxilla *i.e*., the premaxillo-maxillary suture (Fig. 1G).

Morphological changes have been previously associated with the developmental stages of the midpalatal suture^7^, and a similar variation was found in the present study along the rostro-caudal direction of animals of the same age (Fig. 1A–I), indicating a non-synchronous growth of different regions. With regard to the sutural ligament, although the five layers described in the literature^5^ were well-represented in the premaxillary region (Fig. 2I), they were not clearly distinguishable in the more caudal ones, probably because of the progressive reduction of the cambial layers during growth^5^. Although the sutural ligament of the midpalatal suture was described to maintain a distinct stratification even at late growth stages in humans^7^, the highly interlocked palatine region of the swine did not show such layers (Fig. 1A). Suture irregularity, rather than the amount of interdigitation alone, may be better representative of more mature structures.

Furthermore, as already noticed by previous studies^4^, the cambial region presented fibres essentially perpendicular to the surface of the bone, and the middle region of the ligament had orientation more parallel to the sutural margins (Fig. 2H,I). Although such fibre organisation was noticed in the premaxilla, the maxillary region did not show this characteristics (Fig. 2D–F), which were confirmed by SEM imaging showing an unorganised fibrous matrix in the interface (Fig. 3B,C). This net of fibres also presented empty spaces (Fig. 3D), which may facilitate the fluid movement within the ligament during mechanical loading^18^, and potentially contributing to the viscoelastic behaviour reported by previous studies^9,11^.

Although previous histological reports of the midpalatal suture of both humans^4,7,12,13,17^ and animals^5,19^ exist in the published literature, the present work on a swine model provides further details on the anatomical variations along the rostro-caudal direction within the same subject.

### Anatomy

The *Sw* of the maxillary region showed values between 231 μm (SD = 97 μm) and 201 μm (SD = 75 μm) in 18‒38yo humans^17^, which are similar to the data of the same region assessed on the axial (229 µm, IQR = 98 µm) and coronal planes (172 µm, IQR = 59 µm) in the present study. Nevertheless, the premaxillary region (*Sw*_*AX*_ = 1162 µm, IQR = 218 µm; *Sw*_*COR*_ = 1132 µm, IQR = 300 µm) was distinctly wider in the swine model (Fig. 4A,B). Although Knaup *et al*. reported the *Sw* of the midpalatal suture to be similar among different regions in humans^13^, the research summarised values of subjects 18‒68yo, compared to the longitudinal analysis of the present study that revealed a negative correlation between *Sw* and the rostro-caudal gradient (−0.754 on the axial plane, and −0.443 on the coronal plane, p < 0.001). Accordingly, it is worth noting that the length of the palate of the swine is greater than in humans^20^, and this may affect the analysis of the correlation.

The maxillary and palatine regions of the human midpalatal suture develop progressive interdigitation after birth to 18yo^7^, measurable through the interdigitation index (*II*) proposed by Rafferty *et al*.^21^, which corresponds to the *LII*_*COR*_ described in the present study. Beyond the age-dependency, the present work highlighted a region-dependency of the interdigitation. In fact, the suture in the premaxilla was simple (*LII*_*COR*_ = 1.0, IQR = 0.0; *LII*_*SAG*_ = 1.0, IQR = 0.0), whereas the maxillary region (*LII*_*AX*_ = 2.1, IQR = 1.2; *LII*_*COR*_ = 2.9, IQR = 1.5), and the palatine region (*LII*_*AX*_ = 7.3, IQR = 6.2; *LII*_*COR*_ = 4.3, IQR = 1.0) exhibited a greater complexity. Significant differences in *LII* were found among regions (p < 0.001) (Fig. 4C,D), with a positive correlation of *LII* along the rostro-caudal direction in both the axial (+0.718, p < 0.001) and coronal planes (+0.473, p < 0.001). Burn *et al*. analysed with histology the interdigitation of the maxillo-maxillary suture in farm pigs and mini pigs of 18 weeks and 20.5 weeks, respectively^19^, showing changes of sutural complexity in agreement with the present findings. They reported increasing values for the anterior, middle, and posterior maxillary region from 5.08 (SD = 1.75) to 6.88 (SD = 1.54) to 7.66 (SD = 1.62), respectively, in animals exposed to soft diet, and from 3.61 (SD = 0.69) to 6.43 (SD = 1.3) to 7.60 (SD = 1.17), respectively, in animals exposed to hard diet^19^. Perhaps surprisingly, these values were higher than the *LII*_*COR*_ calculated in the present study for older animals, which should be related to greater sutural complexity instead^5,7^. Even so, the sutural length was measured by Burn *et al*. by tracing the margin of the cortical bone on one side of the interface, rather than along the midline of the interface, which may lead to a more intricate and longer line.

Further, discrepancies found between the present study (*LII*_*AX*_ = 2.9) and a previous analysis showing lower sutural interdigitation in humans (*LII*_*AX*_ = 1.4)^22^ might be explained by inter-species differences such as the long maxillary complex of the swine, which can be subjected to greater cantilever forces during mastication^20^. Nevertheless, a previous animal study suggested that sutural interdigitation was not reduced in swine restricted to soft diet^19^, albeit the 12-weeks experimental exposure adopted might have not been long enough to allow macroscopic sutural remodelling.

Despite the fact that growth of the upper face finishes approximately two years before the body height ceases to increase^6^, sutural ossification continues beyond completion of growth^4,12,13,17,22^. With regard to this, data from the coronal plane (*LOI*_*COR*_) of the maxillary region of the midpalatal suture in 15‒35yo humans revealed a greater suture obliteration in its caudal part compared to its rostral^12^. These results are in agreement with the present findings, which showed a positive correlation of *LOI*_*AX*_ with the rostro-caudal gradient (+0.513, p < 0.001). Accordingly, from a visual qualitative evaluation of human dry skulls (*LOI*_*AX*_) probably 11‒68yo, a previous study concluded that the caudal region of the midpalatal suture tends to ossify earlier than its rostral counterpart^23^. Significant differences between these two regions were found in the present study as well, such that the premaxillo-premaxillary suture exhibited no obliteration, and the palato-palatine suture showed obliteration of 5.2% (IQR = 3.9%) (p < 0.001) (Fig. 4E).

Previous studies applied µCT as an attempt to evaluate the *BMD* of the midpalatal suture, and concluded that the sutural bone density could be a parameter limiting maxillary expansion^22^. Besides the fact that maxillary expansion was not performed, and thus no conclusion could be drawn, in the study of Korbmacher *et al*. the *BMD* was measured as the *“ratio of bone volume to total tissue volume”*, which is a simple “bone/non-bone” binary variable. In addition, the study aligned *“the raw dataset on the suture’s midline”* and measured the *BMD* on the respective 2D image corresponding to the sagittal plane of the present study. As a consequence, if an unossified suture is considered (*LOI* = 0.0%), the *BMD* would be close to 0.0% for a straight suture (*LII* = 1.0), which would be visualised as a cross-section of the sutural ligament only, and it would be proportionally increasing with the *LII* because of the intersecting cortical plates. However, the *BMD* should not account for variation of the *LII* and *vice versa*.

Other authors attempted to estimate the response to maxillary expansion through the *“midpalatal suture density ratio”*^24^, by orienting cone-beam computed tomography (CBCT) images on the palatal plane and measuring the properties of a *“6 mm-wide rectangle centered on the midpalatal suture”*. Nevertheless, no calibration was adopted and, even though the study calculated the ratio of the difference of the grayscale-values between the suture and the soft palate, and between the palatal process of the maxilla and the soft palate, the obtained parameter should not be considered representative of the *BMD*. Furthermore, the analysed area included different extents of surrounding bone. All in all, especially for the difficulties in selecting a volume representative of the proper sutural interface, the *BMD* may have little meaning in the evaluation of development and mechanical behaviour of sutures. In fact, the present study revealed no differences between the palatine (*BMD* = 0.67 g/cm^3^, IQR = 0.12 g/cm^3^) and the maxillary region (*BMD* = 0.65 g/cm^3^, IQR = 0.24 g/cm^3^) (p = 0.988), despite the significant differences present in *σ*_*f*_ (p < 0.001), and *E* (p = 0.016). Apparently, other parameters such as the *LOI*, which showed a better agreement with mechanical data, might be more appropriate indicators of sutural maturation.

### Mechanical properties

Bone strain can reach 1000 µm during loading of the facial skeleton in mastication^25^, whilst the opening of the midpalatal suture can be even greater than 5.0 mm during maxillary expansion^26^. Such important deformations of the sutural interface make the mechanical behaviour of the sutural ligament and its surrounding bone of biological and clinical interest. Still, although the midpalatal suture has received histological and anatomical description in humans^4,7,12–14,17,22,23,27^ and animals^5,19,28,29^, and it is the primary structure involved in maxillary expansion in humans^14,24,26^ and animals^3,28,29^, the information regarding its mechanical properties is very sparse and it is probably confined to the analysis of few specimens in animals^30^. This limited knowledge forced authors to design analytical models incorporating values of *E* from studies testing sutures other than the midpalatal^11^, and to develop numerical models based on either arbitrary values^16^ or even representing the suture as an empty space^10^.

In the present study, a decreasing *ε*_*f*_ (−0.691, p < 0.001) and an increasing *E* (+0.494, p < 0.001) were observed toward the caudal region of the suture, depicting a more rigid structure (Fig. 4G–I). Accordingly, an increasing sutural interdigitation (*LII*_*AX*_ + 0.718, p < 0.001; *LII*_*COR*_ + 0.473, p < 0.001) and obliteration (*LOI*_*AX*_ + 0.513, p < 0.001; *LOI*_*COR*_ + 0.725, p < 0.001) were present from rostral to caudal, which is representative of a more interlocked and mature suture.

Furthermore, peculiar mechanical properties arose for each region, suggesting to consider the midpalatal suture to be composed by three segments not only anatomically, but also mechanically. With regard to this, anatomical parameters such as sutural width, interdigitation, obliteration and bone mineral density, resulted to be notably different especially between the premaxilla and the two more caudal regions (Table 1). *ε*_*f*_ followed a similar trend, which might be explained by the greater width of the premaxillo-premaxillary suture, allowing larger soft tissue deformation during bending (Table 1). Whereas, it was the palatine region to be considerably different from the other two in terms of *σ*_*f*_ (Table 1), suggesting that the effect of the amount of obliteration on this parameter might not be linear.

### Analogies with clinical treatments

In pathological conditions such as craniosynostosis *i.e*., the premature ossification of cranial sutures, not only obliteration but also mechanical properties have shown differences between synostosed and healthy sutures in humans^31^. Thus, understanding the amount of physiological ossification is important. Further clinical interest in the ossification of the midpalatal suture is related to the maxillary expansion^27^. For example, in humans, 5.0% of obliteration has been regarded as the limit for splitting the midpalatal sutures, which is not reached in most patients under 25 years of age^12^. Furthermore, a non-parallel V-shaped opening of the maxillary halves has been reported by studies on humans^15,16^, and the rostro-caudal pattern of the mechanical features shown in the present study (Fig. 4) may contribute to formulate research hypothesis about this phenomenon. However, maxillary expansion might be difficult to achieve in some patients despite low LOI^32^. In fact, not only intrinsic sutural properties may be relevant during maxillary expansion, and the hindrance offered by surrounding structures may also restrain the opening of the midpalatal suture in its most caudal area, both in humans^33,34^ and animals^35^.

### Limitations

With regard to the estimation of the mechanical parameters, the use of the cross-section (*S*_*0*_) of the specimen may have influenced the value of *σ*_*f*_. In fact, although the anterior region may have not been significantly affected, due to the low *LII* associated with a relatively flat sutural surface, the most caudal region may have received an over-estimation of the *σ*_*f*_ because of a more extended surface subsequent to a greater interdigitation.

Additionally, *x*_*low*_ was used for the calculation of *ε*_*f*_ and the deformation was assumed to be evenly distributed along the supported length of the specimen. Conversely, utilising *Sw* would have led to the opposite approximation to consider all the *ε*_*f*_ to happen within the sutural ligament and none in the bone. As a consequence, the values provided might be more representative of the entire structure rather than the sutural ligament alone, whose real *ε*_*f*_ might be of greater magnitude. Accordingly, *E* may be also more illustrative of the composite bone-suture-bone structure, instead of one single constituting material.

Further, although the specimens were considered to behave as linear beams, their shape was irregular. Nevertheless, beside some protruding structures such as in the area of the alveolar processes and the projection of the midpalatal suture to join the vomer on the nasal side, the palate of the swine was relatively flat.

In the discussion of the mechanical data it should also be considered that specimens from the premaxilla were cut with a different method compared to the other regions. Nevertheless, all specimens were analysed with µCT and no fracture or damage was present before mechanical testing.

Lastly, animal age was estimated according to local regulations on edible animals and by dental-age assessment, and it should be considered as approximate. This said, a domestic swine reach maturity at about the age of 5–6 months^36^ and a gross inter-species comparison may allow approximately 9 to12-month-old swine to be analogised to young humans at their last growth stages^37^ with completed sutural growth in the maxillary complex. However, associating the age with changes in the sutural parameters, or achieving translational application to clinical treatments, was beyond the scope of the present study.

### Concluding remarks

Despite its clinical relevance in craniofacial development and orthopaedic treatments, hardly any information exists on the mechanical properties of the midpalatal suture, which should be analysed as a composite structure formed by three distinct segments.

The midpalatal suture was characterised by reduced elasticity and increased failure stress proceeding towards its most caudal part. The mechanical findings were supported by concordant anatomical features describing a progressively more mature structure along the same gradient. Nevertheless, the conspicuous anatomical and mechanical distinction of the premaxilla compared and contrasted with the rest of the maxillary complex suggests caution in attributing the highlighted differences only to the sutural maturation, since this structure remains simple and almost unossified throughout swine entire ontogeny^38^.

The present study emphasised the mechano-anatomical topographical variation of the midpalatal suture, on top of the already known age-related changes. These features can be of primary relevance with regard to sutural distraction osteogenesis, and for the understanding of the growth and post-growth modifications of the maxillary complex.

## Methods

### Sample preparation

**Figure 5:**
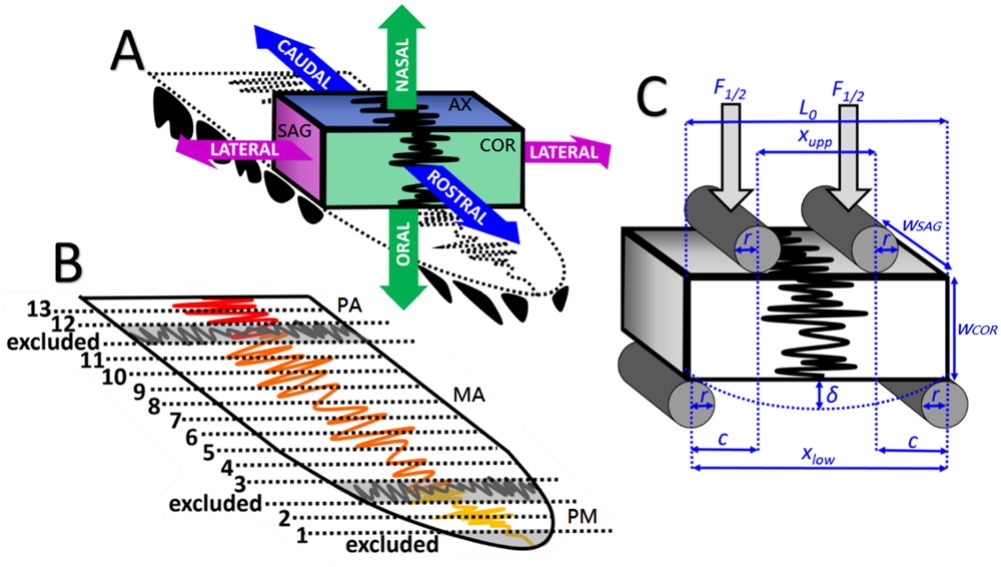
(**A**) Representation of the sagittal plane perpendicular to the oral surface and with rostro-caudal orientation (SAG, violet), the axial plane parallel to the oral surface (AX, blue), and the coronal plane perpendicular to the oral surface and with latero-lateral direction (COR, green). (**B**) Representation of the midpalatal suture: the rostral segment that is the premaxillo-premaxillary suture (PM, yellow), the middle segment that is the maxillo-maxillary suture (MA, orange) and the caudal segment that is the palato-palatine suture (PA, red), which divide the two premaxillae, the two maxillae, and the two palatine bones, respectively. The parts of the palate containing the anterior transversal palatal suture or the posterior transversal palatal sutures (grey), and the most rostral part of the premaxilla (grey) were excluded. Numbers indicate the slices from the most rostral to the most caudal part of the palate. (**C**) Representation of the 4PB test: the jig had a lower span (*x*_*low*_, mm) of 20.00 mm and an upper span (*x*_*upp*_, mm) of 8.00 mm, with *x*_*upp*_ to *x*_*low*_ ratio of 2:5. Rollers had radius (*r*) of 0.75 mm to avoid excessive indentation and stress concentrations in some areas of the specimen (please see Table 2 for explanation of the symbols).

**Figure 6:**
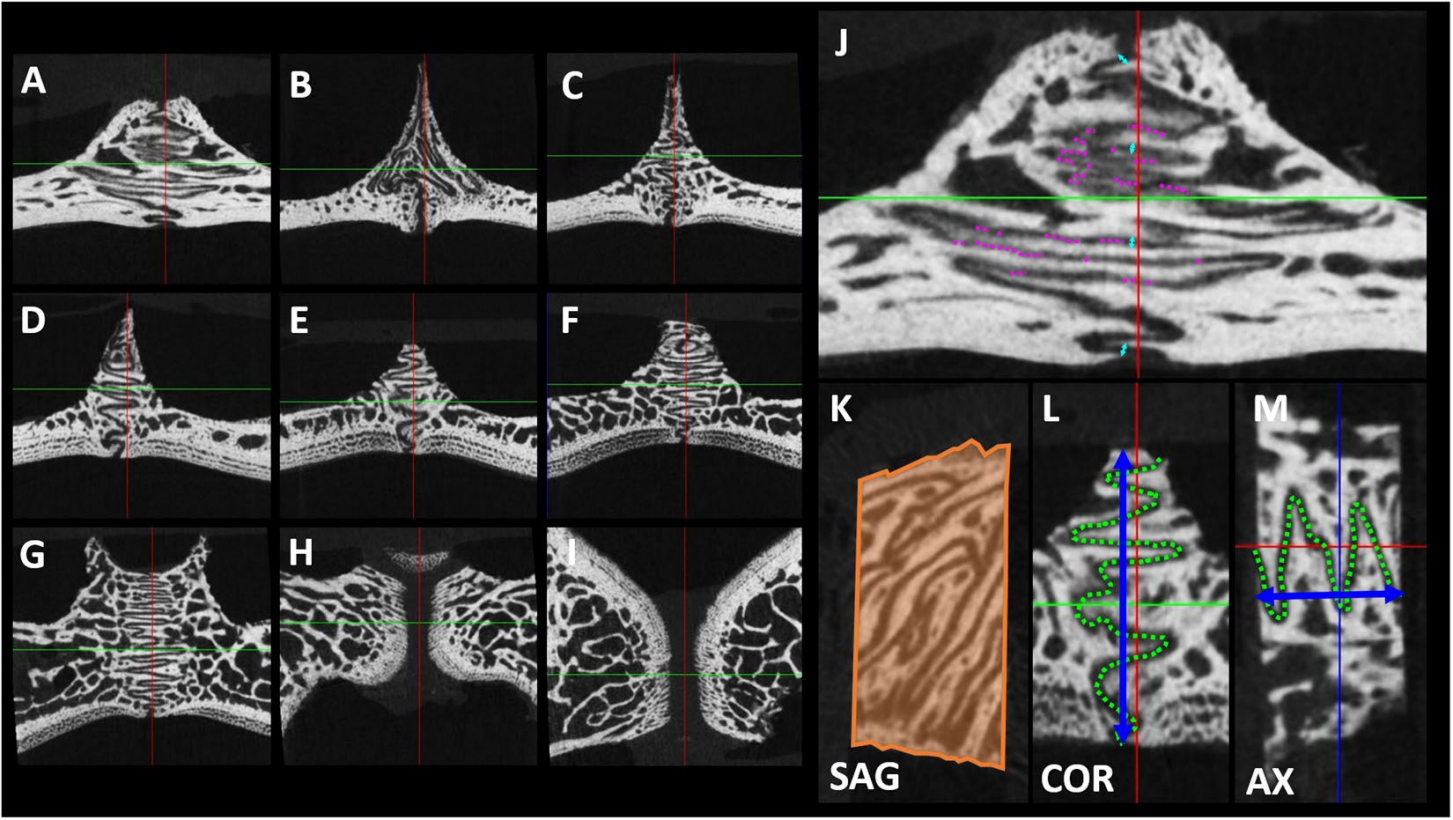
Example images of the µCT parameters. Series of images on the coronal plane from rostral to caudal (**A-I**). On the sagittal plane: cross section of the specimen (*S*_*0*_, orange area), from which a variable oro-nasal width of the specimen can be appreciated (**K**). On the coronal plane: width of the suture (*Sw*_*COR*_, light blue arrow) and obliterated length of the suture (*lob*_*COR*_, dotted violet line) (**J**); oro-nasal width of the specimen (*w*_*COR*_, blue arrow) and length of the suture (*l*_*COR*_, green dotted line) (**L**). On the axial plane: rostro-caudal width of the specimen (*w*_*AX*_, blue arrow) and length of the suture (*l*_*AX*_, dotted green line) (*Sw*_*AX*_ and *lob*_*AX*_ were not represented for simplicity) (**M**).

Animals were purchased from the market after slaughter for human consumption, in agreement with regulations by the Committee on the Use of Live Animals in Teaching and Research (CULATR) of The University of Hong Kong. Eleven palates were harvested with high-speed hand-piece from swine heads (approximately 9 to 12-month-old) under water irrigation. One palate was used for histology, and ten for µCT and 4-point-bending (4PB). Samples were sliced parallel to the coronal plane (Fig. 5) via cutting-machine (IsoMet 5000, Buehler^©^, USA), under water irrigation. Specimens from the premaxilla could not be cut via automatic cutting machine and were sliced with a circular hand saw (9710, Robust^©^, Hong Kong) under water irrigation. According to its position in the palate, the width of the specimen between its right and left extremity (*w*_*AX*_, mm) and the width between its nasal and oral extremity (*w*_*COR*_, mm) were left according to the anatomy, whereas the width between its rostral and caudal extremity (*w*_*SAG*_, mm) (Fig. 6) was standardised (≈2.5 mm). Alternate specimens were selected for µCT and 4PB, keeping the remaining for SEM (VP-SEM SU1510, Hitachi^©^, Japan). Thirteen specimens were tested from each palate (Fig. 5B). Specimens were immersed in saline solution (0.9% NaCl), refrigerated at 4 °C, and evaluations were performed within 48 h from the death of the animal.

**Table 2:** List of the variables.

symbol	list of the variables	
	name	unit of measurement
*F*	force	N
*δ*	deflection	mm
*r*	radius of the roller	mm
*c*	horizontal distance between upper and lower roller on one side	mm
*x*_*upp*_	upper span length	mm
*x*_*low*_	lower span length	mm
*l*	length of the suture	mm
*w*	width of the specimen	mm
*W*	length of the beam	mm
*S*_0_	cross-section of the specimen	mm^2^
*lob*	obliterated length of the suture	mm
*E*	elastic modulus	Pa
*σ*	stress	Pa
*ε*	strain	mm/mm
*Sw*	width of the suture	µm
*LII*	linear interdigitation index	mm/mm
*LOI*	linear obliteration index	mm/mm
*Pos*_*CAT*_	region of the specimen	PM, MA, PA
*Pos*_*CON*_	position of the specimen	1 to 13

### Histology

One palate was fixed with 4% paraformaldehyde (HO(CH_2_O)_n_H) for 24 h. After decalcification with formic acid (HCOOH) for five days, ≈3.0 mm slices were prepared using a surgical blade on the coronal plane. Auto tissue processing (Excelsior ES, Thermo Scientific^©^, UK) was applied following standard steps in ethanol and xylene. Specimens were embedded in paraffin wax and cut into 6 µm sections using a microtome (RM 2155, LEICA^©^, Germany) on the same plane. Sections were stained with haematoxylin (Sigma H9627) and eosin (Merck 15935), and mounted with a mounting medium (Fisher Scientific, SP15–500, Permount^©^). Images were captured with an optical microscope (Eclipse LV 100 POL, Nikon^©^, Japan).

### µCT

µCT scans (SkyScan^©^1172, Bruker, US) were acquired at 640 × 512 pixel resolution, 80 kV voltage, 100 mA current, 1° rotation step, and 25 μm pixel size. Two standardised phantoms of hydroxyapatite (Ca_5_(PO_4_)_3_(OH)) with bone mineral density (*BMD*, g/cm^3^) of 0.25 g/cm^3^ and 0.75 g/cm^3^ were simultaneously scanned in each acquisition. *BMD* calibration was obtained for each specimen from the average attenuation coefficient of a volume of one-hundred layers of the µCT of each phantom and a round region of interest with 4.0 mm diameter. *BMD* was calculated for a cylinder of 6.0 mm length and 1.0 mm diameter oriented on the latero-lateral axis (CTAnalyser^©^, Bruker, US) after aligning the specimen (DataViewer^©^, Bruker, US). The length of the suture (*l*, mm) *i.e*., the line traced along the midline of the sutural interface, was measured on both planes (axial and coronal) (Fig. 6). The linear interdigitation index (*LII*, mm/mm) *i.e*., the ratio between the length of the suture and the width of the specimen, was calculated for both planes (axial and coronal):

1 $$LI{I}_{AX}={l}_{AX}/{w}_{AX}$$

and

2 $$LI{I}_{COR}={l}_{COR}/{w}_{COR}$$

The sutural width (*Sw*, µm) *i.e*., the shortest distance betw/een the two cortical plates enclosing the sutural ligament, was calculated as the average among four equidistant measurements along the suture on both planes (axial and coronal) (Fig. 6). The obliterated length of the suture (*lob*, mm) *i.e*., the sum of the obliterated segments along the suture (where obliterated means that no radiotransparent space was present between the two cortical plates) (Fig. 6), was calculated on both planes (axial and coronal). The linear obliteration index (*LOI*, mm/mm) *i.e*., the ratio between the length of obliterated suture and the total length of the suture, was calculated for both planes (axial and coronal):

3 $$LO{I}_{AX}=lo{b}_{AX}/{w}_{AX}$$

and

4 $$LO{I}_{COR}=lo{b}_{COR}/{w}_{COR}$$

The surface of the cross-section of the specimen (*S*_*0*_, mm^2^) was measured on the sagittal plane (Fig. 6). Anatomical parameters were measured with graphical software (ImageJ^39^).

### Four-point-bending

4PB tests till failure were performed using a testing machine (Instron^©^ 4444, UK) with a ±100 N load cell (Fig. 5C). Specimens were positioned with the oral side facing the lower span (*x*_*low*_), similar to the bending during maxillary expansion, and loaded at 1.00 mm/min at room temperature (≈25 °C), while kept moist with saline solution spray. Data were acquired at 0.1 KHz recording deflection (*δ*, mm) and force (*F*, N). Specimens were assumed to be solid linear beams with a constant cross-section (*S*_*0*_). After 4PB, specimens failed along the suture were identified with an optical stereo-microscope (UFX-II, Nikon^©^, Japan) at 40× magnification^40^. Only these specimens were included in the mechanical calculation and only geometry at the failure site was considered (*S*_*0*_ = *S*_*0f*_, *w*_*AX*_ = *w*_*AXf*_, and *w*_*COR*_ = *w*_*CORf*_). Because of the static bending, the mass of the beam was assumed to be negligible and only *x*_*low*_, and not the actual beam length (*W*), was considered. Because of a variable anatomy on the oro-nasal direction (Fig. 6K), a mathematical *w′*_*COR*_ rather than the actual µCT measurement (*w*_*COR*_) was used to better comply with the assumption of uniform *S*_*0*_:

5 $${{w}^{\prime} }_{{C}{O}{R}}=\frac{{{S}}_{{0}}}{{{w}}_{{A}{X}}}$$

The elastic modulus (*E*, N/m^2^) was calculated:

6 $$E=\frac{{F}_{f}}{{\delta }_{f}}\frac{\,2c(3{{x}_{low}}^{2}{\textstyle \text{-}}4{c}^{2})\,}{8{w}_{AX}{{{w}^{\prime} }_{COR}}^{3}}$$

where *δ*_*f*_ (mm) is the deflection at failure.

Failure strain (*ε*_*f*_, mm/mm), at ½ *x*_*low*_ and ½ w_COR__*f*_, was calculated:

7 $${\varepsilon }_{f}=\frac{12{{w}^{\prime} }_{COR}}{(3{{x}_{low}}^{2}{\textstyle \text{-}}4{c}^{2})}{\delta }_{f}$$

Failure stress (*σ*_*f*_, N/m^2^) was calculated:

8 $${\sigma }_{f}={F}_{f}\frac{3{\rm{c}}}{{w}_{AX}{{{w}^{\prime} }_{COR}}^{2}}$$

where *F*_*f*_ (N) is the force at failure.

### Data analysis

Categories were created for the premaxillary (PM), maxillary (MA), and palatine (PA) region *i.e*., (*Pos*_*CAT*_), and specimens were also numbered from rostral to caudal (from 1 to 13) *i.e*., (*Pos*_*CON*_). Anatomical parameters (*Sw*, *LII*, *LOI*, *BMD*) were calculated including all the data, whereas mechanical parameters (*E*, *ε*_*f*_, *σ*_*f*_) were calculated including only data from specimens failed along the suture. Normality of the data distribution was assessed with the Shapiro-Wilk test. The Kruskal-Wallis one-way ANOVA with the Mann-Whitney’s *post hoc* test were used to compare *E*, *ε*_*f*_, *σ*_*f*_, *Sw*, *LII*, *LOI*, and *BMD* relatively to *Pos*_*CAT*_, and the Spearman’s rank coefficient was used to analyse correlations with *Pos*_*CON*_. Data analysis was performed with statistical software (SPSS^©^ V23.0, IBM, US) at significance level α = 0.05.

The datasets generated during and/or analysed during the current study are available from the corresponding author on reasonable request.
